# Multiorgan Detection and Characterization of Protease-Resistant Prion Protein in a Case of Variant CJD Examined in the United States

**DOI:** 10.1371/journal.pone.0008765

**Published:** 2010-01-19

**Authors:** Silvio Notari, Francisco J. Moleres, Stephen B. Hunter, Ermias D. Belay, Lawrence B. Schonberger, Ignazio Cali, Piero Parchi, Wun-Ju Shieh, Paul Brown, Sherif Zaki, Wen-Quan Zou, Pierluigi Gambetti

**Affiliations:** 1 Institute of Pathology, Case Western Reserve University, Cleveland, Ohio, United States of America; 2 Department of Pathology and Laboratory Medicine, Emory University Hospital, Atlanta, Georgia, United States of America; 3 National Center for Zoonotic, Vector-Borne, and Enteric Diseases, Center for Disease Control and Prevention, Atlanta, Georgia, United States of America; 4 Dipartimento di Scienze Neurologiche, Universita' di Bologna, Italy; 5 CEA/DSV/iMETI/SEPIA, France; National Institute for Infectious Diseases L. Spallanzani, Italy

## Abstract

**Background:**

Variant Creutzfeldt–Jakob disease (vCJD) is a prion disease thought to be acquired by the consumption of prion-contaminated beef products. To date, over 200 cases have been identified around the world, but mainly in the United Kingdom. Three cases have been identified in the United States; however, these subjects were likely exposed to prion infection elsewhere. Here we report on the first of these subjects.

**Methodology/Principal Findings:**

Neuropathological and genetic examinations were carried out using standard procedures. We assessed the presence and characteristics of protease-resistant prion protein (PrP^res^) in brain and 23 other organs and tissues using immunoblots performed directly on total homogenate or following sodium phosphotungstate precipitation to increase PrP^res^ detectability. The brain showed a lack of typical spongiform degeneration and had large plaques, likely stemming from the extensive neuronal loss caused by the long duration (32 months) of the disease. The PrP^res^ found in the brain had the typical characteristics of the PrP^res^ present in vCJD. In addition to the brain and other organs known to be prion positive in vCJD, such as the lymphoreticular system, pituitary and adrenal glands, and gastrointestinal tract, PrP^res^ was also detected for the first time in the dura mater, liver, pancreas, kidney, ovary, uterus, and skin.

**Conclusions/Significance:**

Our results indicate that the number of organs affected in vCJD is greater than previously realized and further underscore the risk of iatrogenic transmission in vCJD.

## Introduction

Variant Creutzfeldt-Jakob disease (vCJD) was first reported in 1996 as a novel non-inherited form of prion disease [1]. Although the disease bears some of the classical features of the sporadic form of the human transmissible spongiform encephalopathies (TSE) or prion diseases, it has distinctive features [2]–[4]. Most remarkably, vCJD presents at an average age of 26 years [4]; histopathologically is characterized by the presence of plaques containing prion protein surrounded by vacuoles referred to as “florid” or “daisy” plaques [1]. Furthermore, the abnormal and pathogenic prion protein isoform (hereafter identified as PrP^res^) associated with vCJD has features that are unique among non-inherited human prion diseases [5]. PrP^res^ is thought to be the major or lone component of the infectious agent of prion disease, the so called “prion”. However, occasionally not direct correlation has been reported between PrP^res^ and infectivity [6].

The distinctive features of vCJD, along with its detection in the UK following the peak of the British epidemic of the prion disease bovine spongiform encephalopathy (BSE), pointed to the consumption of prion-contaminated beef products as the possible source of infection [1], [7]. Successful transmission to non-human primates and transgenic mice expressing the human prion protein (human PrP) with replication of major features of the vCJD phenotype, provided overwhelming evidence supporting the notion of cattle-to-human transmission [8]–[10]. These findings established vCJD as the first Western world prion disease to be acquired by oral infection. Kuru, discovered in the 1950s, was endemic among New Guinea tribes practicing ritualistic cannibalism [11]–[13].

The oral route of prion infection in vCJD raised the possibility that tissues and organs, beside the central nervous system (CNS), might also be affected. To date, PrP^res^ has been reported in several tissues and organs outside the CNS of vCJD patients (Table 1) [14]–[19, P. Brown, unpublished data].

**Table 1:** Peripheral tissues shown to contain PrP^res^ in vCJD^1^.

Adrenal gland	Pituitary gland
Appendix	Rectum
Autonomic ganglia	Retina
Blood vessels	Skeletal muscle
Colon	Spinal ganglia
Ileum	Spleen
Jejunum	Thymus
Lymph nodes	Tonsil
Optic nerve	Trigeminal ganglia
Peripheral nerves	

1 Data obtained from immunoblotting and immunohistochemistry studies. Brown P., unpublished data; WHO Expert Committee Meeting, Baden, Austria, 18 May 2007 (updated through Jan 2009).

Although the amount of PrP^res^ in non-neural tissues is small compared to that in the brain, the risk posed by the spread of even small amounts of PrP^res^ has been underscored by the iatrogenic transmission of vCJD from blood donors in the preclinical phase of the disease [20].

We examined the main characteristics and tissue distribution of PrP^res^ in a case of vCJD, in which the disease was most likely acquired in the UK but which is officially referred to as an American case because illness onset occurred in the US [21]. In an extensive autopsy examination, sodium phosphotungstate (NaPTA) precipitation, a highly sensitive method of PrP^res^ detection [14], [22], was used to establish the presence and estimate the relative amounts of PrP^res^ in several organs and tissues made available to the National Prion Disease Pathology Surveillance Center (NPDPSC).

## Materials and Methods

### Collection and Processing of Tissues

A whole body autopsy was performed within 20 hours from death. The National Prion Disease Pathology Surveillance Center (NPDPSC) received frozen and fixed tissue samples. Frozen tissue included slices from one cerebral and cerebellar hemisphere, portions of pituitary gland and dura mater as well as samples from the trachea, breast, heart, lung, esophagus, stomach, duodenum, jejunum, ileum, colon, liver, spleen, pancreas, adrenal gland, kidney, urinary bladder, uterus, ovary, mesenteric lymph nodes, diaphragm and skin. The skin was taken from the chest wall. In addition, paraffin blocks or sections from the same tissues were also received. Frozen tissues were stored at −80°C.

### Histopathology and Prion Protein Immunohistochemistry

Histology and immunohistochemistry were carried out as previously described [23] on brain sections from frontal, temporal and parietal neocortices (the occipital cortex was unavailable), neo-striatum, thalamus, cerebellar hemisphere and on sections from all received tissues. Immunohistochemistry was carried out with the monoclonal antibody 3F4 to the PrP residues 109–112 [24].

### Genetic Analysis

Genotyping was performed on genomic DNA extracted from blood as previously described [25].

### Preparation of Tissue Homogenates

Tissue homogenates (TH) (10%, wt/vol) were prepared at 4°C in phosphate buffered saline (PBS) lacking Ca^2+^ and Mg^2+^, 1% Sarkosyl (pH 7.4), followed by centrifugation at 1000×*g* for 5 minutes to remove cellular debris. Excess of collagen was eliminated by homogenizing tissue and removing the white and dense fraction containing mainly collagen from the fraction rich in parenchymal tissue. Contamination of non-nervous tissue with brain tissue that might have occurred at autopsy was controlled by sampling the depth of the organs and discarding the tissue at the surface. Dura mater where this procedure was unsuitable was rinsed extensively with PBS before homogenization.

### Sodium Phosphotungstate Precipitation (NaPTA)

Precipitation with NaPTA was carried out according to Wadsworth et al [14] with minor modifications. Briefly, 100 mg of wet tissue were homogenized (10% wt/vol) with PBS lacking Ca^2+^ and Mg^2+^, 2% Sarkosyl (pH 7.4), followed by centrifugation at 1000×*g* for 5 minutes to remove cellular debris. A fraction of the supernatant was collected and frozen for immunoblot analysis, whereas a second fraction of 500 µl was mixed with an equal volume of PBS prepared as above. Samples were adjusted to a final concentration of 50 units/ml of Benzonase and 1 µM of MgCl_2_ and incubated at 37°C for 30 minutes, followed by the addition of 81.3 µl of a pre-warmed solution containing 4% NaPTA and 170 mM MgCl_2_. After incubation for another 30 minutes at 37°C and constant agitation, samples were centrifuged at 16,000×g for 30 minutes. The supernatant was discarded whereas the pellet was re-suspended in 200 µl of PBS containing 0.1% Sarkosyl (pH 7.4) with the addition of 50 µl EDTA 250 mM (pH 8), in order to remove the white precipitate present in solution. After an additional centrifugation at 16,000×g for 30 minute, supernatants were discarded and the pellets re-suspended in 30 µl of PBS containing 0.1% Sarkosyl.

#### Immunoblot

Aliquots of TH or NaPTA-precipitated samples were either examined untreated or after treatment for 1 hour at 37°C with proteinase K (PK) (specific activity 44 units(U)/mg, Sigma Aldrich) at the concentration of 2 U/ml (1 U/ml corresponds to 23 µg/ml when PK specific activity is 44 U/mg) for 60 minutes at 37°C while constantly agitated. The reaction was terminated by addition of 3 mM of phenylmethylsulfonyl fluoride. Samples were diluted in sample buffer (final concentration: 3% sodium dodecyl sulfate [SDS], 4% ß-mercaptoethanol, 10% glycerol, 2 mM EDTA, 62.5 mM Tris, pH 6.8) and boiled for 10 minutes before loading. For deglycoyslation of the protein, samples were denatured and incubated in the presence of recombinant peptide N glycosidase F (PNGase F) according to the manufacturer's protocol (New England Biolabs). Protein samples were separated in 15% Tris-Glycine SDS-PAGE gels using gel electrophoresis apparatus holding running gels of different lengths (Criterion 7 cm and home made 15 cm high-resolution system, Bio-Rad). Proteins were transferred to Immobilon P (Millipore) for 2 h at 65 V, blocked in 5% (*w/v*) non-fat milk powder in TBS containing 0.1% (v/v) Tween-20 (TBST) (blocking solution), and incubated overnight at 4°C with selected antibodies. After several washes in TBST, membranes where incubated with a 1∶4,000 dilution of a peroxidase-conjugated secondary antibody in TBST for 60 minutes at room temperature, washed in TBST and visualized by enhanced chemiluminescence (Amersham ECL Plus, GE Healthcare) on Kodak BioMax XAR films (Eastman Kodak). Two antibodies to human PrP were used: the monoclonal antibody 3F4 (to residues 109–112) and the rabbit antisera 2301 (to residues 220–231).

### Ethics Statement

This study was conducted according to the principles expressed in the Declaration of Helsinki. No Institutional Review Board review was required because federal regulations do not require approval of research on deceased patients by the Board. Written informed consent for use of patient information/tissue specimens for research purposes has been obtained.

## Results

### Clinical History

Clinical data on the present patient have been reported in detail [21]. Briefly, the patient lived in Britain until the age of 13 and immigrated to the US in 1992. In early November 2001, at the age of 22 years, the patient was evaluated for depression, emotional instability and memory loss, followed one month later by involuntary movements, gait disturbances and incontinence. During the ensuing three months, the patient's motor and cognitive deficits worsened, and confusion, hallucination, dysarthria, bradykinesia, and spasticity also occurred. The diagnosis of vCJD was made following brain magnetic resonance imaging and confirmed by immunoblot and immunohistochemistry of tonsil tissue. She received an experimental treatment with quinacrine for 3 months, but showed only minimal and transitory improvement. The patient died in June 2004, 32 months after the clinical onset.

### Histopathological Examination

**Figure 1:**
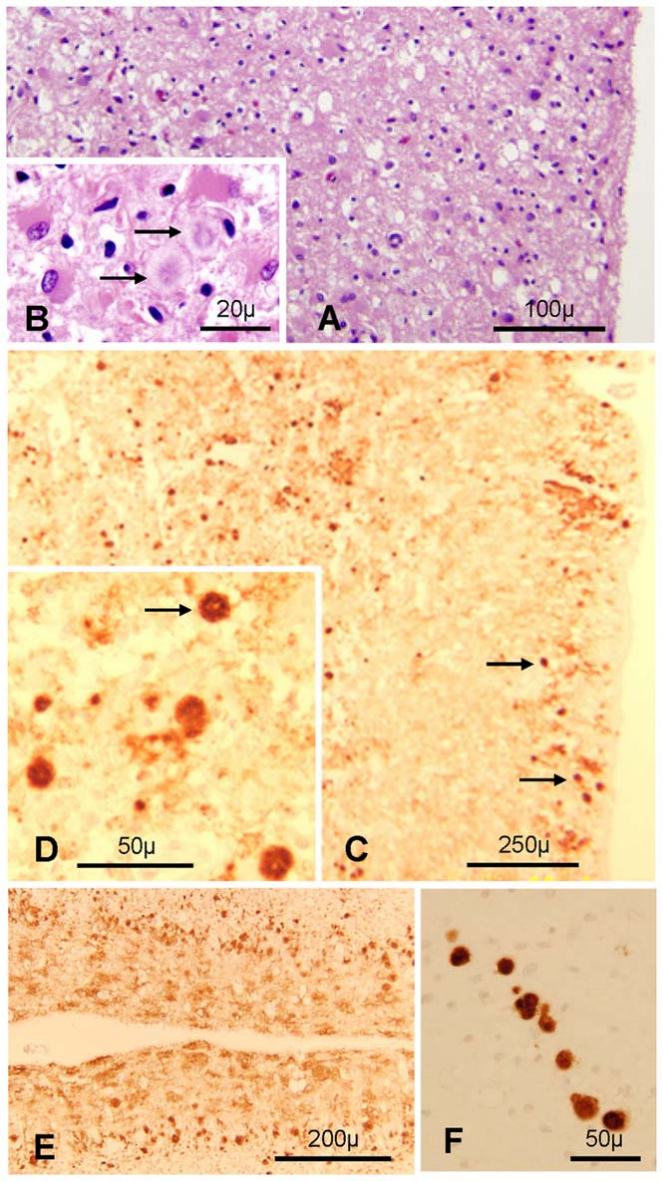
Histological and immunohistochemical features. **A** and **B**. The cerebral cortex is markedly disrupted with prominent astrogliosis and presence of amyloid plaques (arrows in **B**); frontal cortex; H.E. **C** and **D**: PrP immunostaining shows the presence of many plaques (arrows) and plaque-like aggregates in superficial and deep cortical regions with a punctate background staining; frontal cortex. **E**: Distinctive spot-like PrP immunostaining in the molecular layer and many plaques especially evident in the Purkinje cell layer of the cerebellum. **F**: Plaques, often in a row are present in the superficial white matter; cerebellum. **C–F**: Monoclonal antibody (mAb) 3F4.

Both gray and white matter structures were severely atrophic with nearly total loss of neurons and replacement of the neuropil with prominent gemistocytic astrogliosis (Fig. 1A and B). Thus, the typical spongiform degeneration was not observed. Instead, there were irregular extracellular spaces consistent with the astroglial scarring present in the cerebral cortex (Fig. 1B). Macrophages were also present occasionally, especially in the white matter, and probably reflected Wallerian degeneration. The cerebral and cerebellar cortices and the basal ganglia were more affected than the thalamus. Many mono-centric plaques, often large and occasionally surrounded by “pseudo vacuoles”, were present preferentially in the deep cerebral cortex and superficial white matter as well as, to a lesser extent, in the cerebellar cortex and white matter (Fig. 1B). All the organs that had been examined (see Material and Methods for details) were unremarkable except for the kidney and the descending colon which evidenced lymphocytic inflammatory infiltrates (data not shown). In the kidney the infiltrates displayed a focal follicular pattern consistent with interstitial nephritis whereas in the descending colon the lymphocytic infiltrates were linear and located in the sub-mucosa.

Immunohistochemical staining for PrP of brain sections revealed numerous well circumscribed as well as more diffuse PrP deposits consistent with unicentric plaques or early plaques (also called plaque-like) formations, which were especially prominent in the very superficial and deep cortical layers (Fig. 1C and D). Granular and “synaptic” immunostaining patterns were easily detectable in basal ganglia and thalamus. The cerebellum showed a leopard skin-like immunostaining and plaque-like patterns in the molecular and granule cell layer, respectively (Fig. 1E and F). Polarized light examination confirmed that the plaques contained amyloid (data not shown). No PrP immunostaining was detected in any of the tissues examined outside the brain.

**Genetic analysis** demonstrated methionine homozygosity at codon 129 and no mutations or other variations in the open reading frame of the PrP gene.

### Characterization of Brain PrP

**Figure 2:**
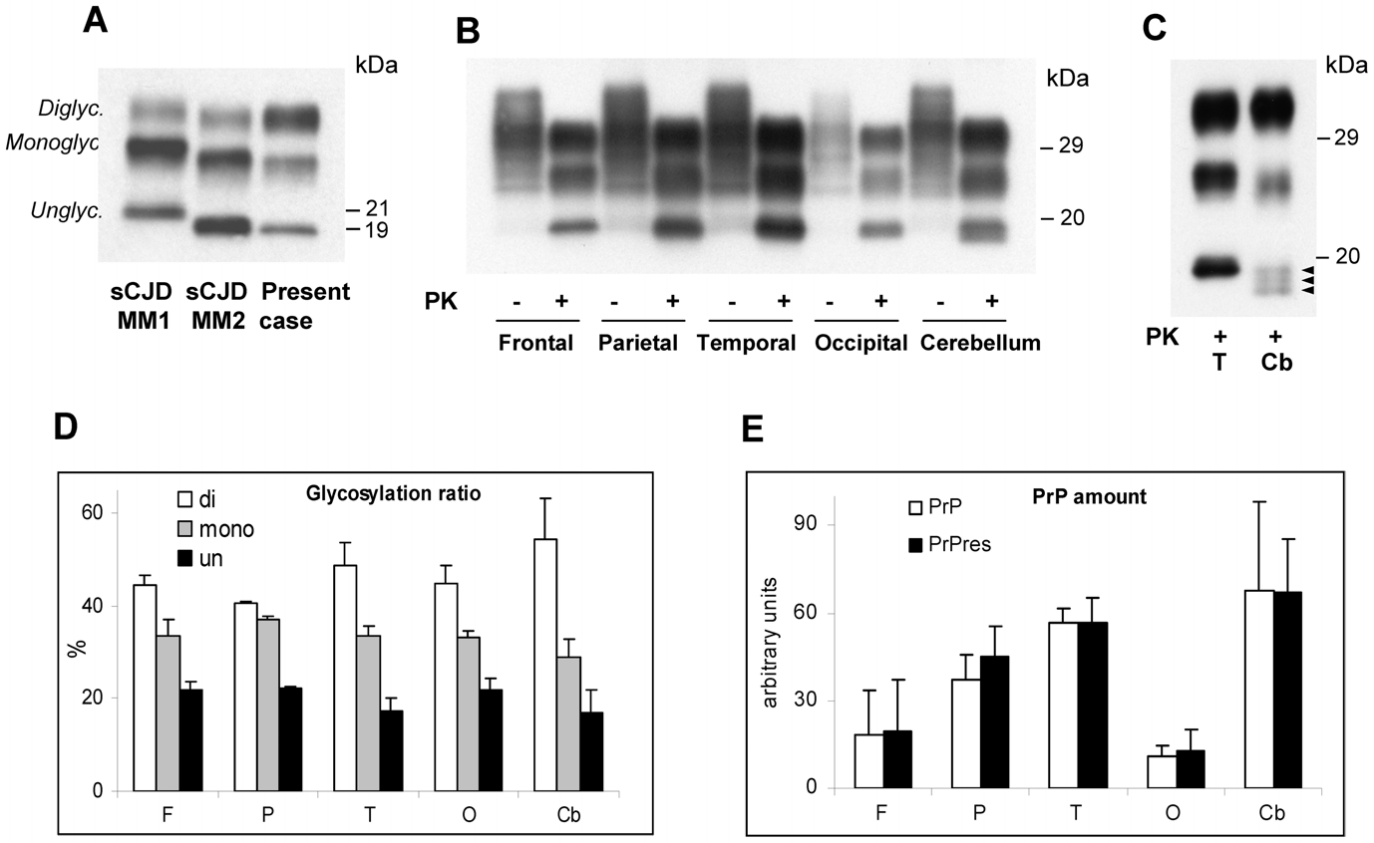
Detection and characterization of PrP^res^ and PK-sensitive PrP in brain. **A:** Immunoblot of total homogenates (TH), treated with proteinase K (PK), obtained from the frontal cortex of sCJDMM1, sCJDMM2 (representing PrP^res^ types 1 and 2, respectively) and the present case showing the over-representation of the upper band (Diglyc.) containing the diglycosylated form, and the co-migration of the lowest band (Unglyc.), containing the unglycosylated form, with the corresponding band of sCJDMM2. **B:** Immunoblot of TH from the four regions of the cerebral cortex and the cerebellum, treated with PK as indicated. The cerebellar unglycosylated PrP^res^ isoform generates a thicker and overall slightly faster migrating band than the corresponding PrP^res^ from the cerebral cortex. **C:** A high-resolution immunoblot (15%, 15 cm long gel) confirms that the monoglycosylated and unglycosylated PrP^res^ isoforms from the cerebellum have a faster electrophoretic mobility than the corresponding forms from the cerebral cortex, and shows that the cerebellar unglycosylated isoform resolves into three fragments including a 19 kDa band, corresponding to PrP^res^ type 2, and two additional bands of slightly lower relative molecular weight (arrowheads); T: Temporal; Cb: Cerebellum. In **A–C** membranes were probed with the mAb 3F4. **D** and **E:** Ratios of the PrP^res^ glycoforms (D) and of the total PrP and PrP^res^ (E) obtained from the same brain regions examined in panel B. Each bar represents the mean ± SD of three densitometric determinations on each of two tissue samples.

Immunoblot analyses of the PK-digested total homogenate (TH) from all cerebral cortices examined displayed the characteristic electrophoretic mobility and glycoform ratios of the PrP^res^ described in vCJD (Fig. 2A) [5], [26]. In the cerebellum PrP^res^ showed a slightly faster migration (Fig. 2B). When a high resolution gel (15%, 15 cm long) was used, the PrP^res^ unglycosylated form in the cerebellum appeared to resolve into three bands, which included the band corresponding to the PrP^res^ type 2 of 19 kDa and two additional bands that migrated about 0.5 kDa and 1 kDa faster (Fig. 2C). The upper band containing the diglycosylated PrP isoform was over-represented in all brain regions examined including the cerebellum (Fig. 2B and D). Total PrP and PrP^res^ were best represented in the temporal cortex and cerebellum while they were present in the least amount least amount in the occipital cortex (Fig. 2B and E). In addition, we confirmed the presence of a 17 kDa PrP^res^ fragment matching the anchorless PrP^res^ type 2 fragment previously described in sporadic CJD (sCJD) and vCJD [27], whereas the 12/13 C-terminal fragment commonly present in sCJD was not detected (data not shown) [28]. These two findings are in agreement with the previously reported molecular characteristics of PrP^res^ from vCJD [27]. To assess whether PrP^res^ types 1 and 2 co-occurred in brain as previously reported [29], we digested the TH with a high concentration (32 U/ml) of PK and used high resolution gels (15%, 15 cm long), a technique that allows for the detection of even small amounts of PrP^res^ type 1 and 2 (up to 3–5% of total PrP^res^) when they co-exist [30]. This procedure failed to demonstrate PrP^res^ type 1 in the brain regions examined in this case (data not shown).

### Detection of PrP^res^ in Non-Nervous Tissues

**Figure 3:**
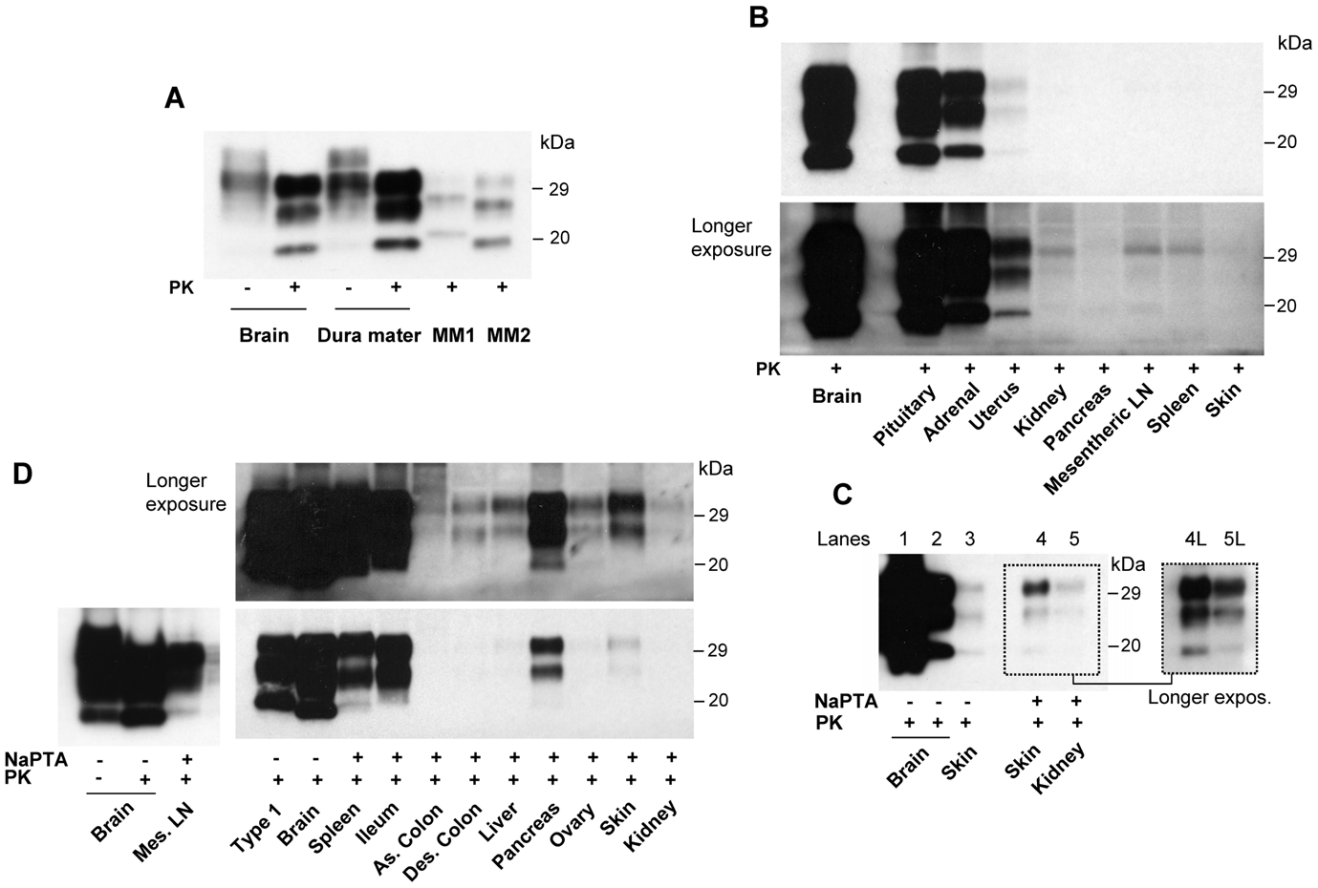
Detection of PrP^res^ in non-nervous tissues. **A:** PrP^res^ from dura mater and frontal cortex (1∶24 dilution) from the present case is compared to PrP^res^ of the frontal cortex from sCJDMM1 (type 1) and sCJDMM2 (type 2). **B:** Two film exposures of immunoblots from non-nervous tissues compared with that of the frontal cortex (1∶10 dilution). Pituitary gland, adrenal gland and uterus are clearly positive while the bands in the remaining preparations are considered to be non specific. **C:** PrP^res^ from skin (double TH loading, equivalent to 4 mg of wet tissue) is barely detectable in TH (Lane 3) compared with frontal cortex TH, which is diluted 1∶4 (lane 1) or 1∶130 (lane 2). Skin PrP^res^ is better detectable along with the kidney PrP^res^ after sodium phosphotungstate (NaPTA) precipitation of PrP^res^ (Lanes 4 and 5), especially after long exposure (Lanes 4L and 5L) (kidney TH loaded in double amount; probed with mAb 3F4). **D:** PrP^res^ from mesenteric lymph nodes and other visceral organs recovered following NaPTA precipitation and compared with TH from the frontal cortex (1∶120 dilution) and sCJDMM1 following two film exposures. All organs but ascending colon are positive. Of note, unglycosylated isoform is underrepresented in all NaPTA precipitated samples as compared with that of the TH preparations (see panel A, lanes 3 and 4 and panel B). **A–D:** Membranes were probed with the mAb 3F4.

PrP^res^ could be easily detected in the dura mater, the pituitary and adrenal glands, and the uterus using direct blotting of the TH (Table 2 and Fig. 3A and B). Detection in the skin required doubling the TH concentration (equivalent to 4 mg of wet tissue) but this procedure failed to reveal PrP^res^ in other organs (Fig. 3B, 3C, and data not shown).

**Table 2:** Synopsis of PrP^res^ analyses in the brain and other tissues.

POSITIVE TISSUES	TH^2^	NaPTA^3^	PrP^res4^	Glyc. ratio	NEGATIVE TISSUES
Brain	**+**	**+**	100	47∶33∶20	
**Other Tissues**					
***Dura mater^1^***	**+**	**+**	8.6	53∶34∶13	Asc. Colon
Pituitary gland	**+**	**+**	5.3	40∶41∶19	Breast
Adrenal gland	**+**	**+**	2.5	45∶39∶17	Diaphragm
***Uterus***	**+**	**+**	6·10^−1^	50∶38∶13	Duodenum
***Skin***	**+**	**+**	7·10^−2^	39∶43∶19	Esophagus
Spleen	**-**	**+**	<7·10^−2^	60∶35∶6	Heart
Ileum	**-**	**+**	<7·10^−2^	57∶40∶3	Jejunum
Mesenteric LN	**-**	**+**	<7·10^−2^	48∶46∶11	Left lung
***Pancreas***	**-**	**+**	<7·10^−2^	53∶36∶11	Right lung
***Liver***	**-**	**+**	<7·10^−2^	57∶34∶9	Stomach
***Ovary***	**-**	**+**	<7·10^−2^	58∶37∶5	Trachea
***Descending colon*** **^5^**	**-**	**+**	<7·10^−2^	62∶34∶5	Urinary bladder
***Kidney***	**-**	**+**	<7·10^−2^	52∶35∶13	

1 In ***bold italic*** organs where PrP^res^ had not been detected previously.

2 TH: PrP^res^ searched in tissue homogenate.

3 NaPTA: PrP^res^ searched following enrichment with sodium phosphotungstate precipitation.

4 Amount of PrP^res^ expressed as percentage of PrP^res^ present in the frontal cortex. Glyc. ratio: glycoform ratio expressed as percentage of the sum of the three isoforms and representing diglycosylated:monoglycosylated:unglycosylated forms. Data listed for tissue positive in both TH and NaPTA preparations were obtained from TH.

5 PrP^res^ was previously reported in colon with no specification of the segment examined.

With NaPTA precipitation, we easily detected PrP^res^ in the mesenteric lymph nodes, spleen, ileum, pancreas, skin, and to a lesser extent, in the descending colon, liver, ovary and kidney (Table 2 and Fig. 3D). The unequivocal identification of PrP^res^ in the kidney required multiple sampling and an additional two-fold loading of the gel but these procedures failed to reveal PrP^res^ in the ascending colon (Fig. 3C and D). Compared to direct TH blotting, NaPTA preparations often revealed a slower electrophoretic migration of up to 0.5 kDa (Fig. 3C, 3D and data not shown), as previously reported [14].

A significant over-representation of the diglycosylated form with a ratio comparable to that of the brain was apparently maintained in all the organs except the pituitary gland, the skin and some of the TH preparations from the uterus where diglycosylated and monoglycosylated isoforms had nearly the same concentration (Table 2, Fig. 3). Generally, in the NaPTA preparations the unglycosylated form was less well represented than in the TH preparations (Table 2, Fig. 3 and data not shown).

**Figure 4:**
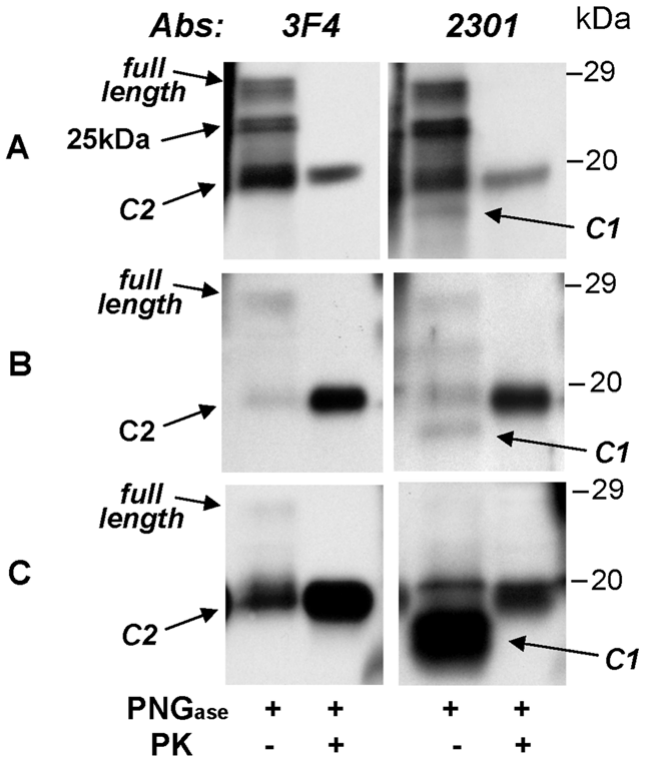
Characteristics of PK-sensitive PrP. Immunoblot analysis of total homogenate from brain, pituitary gland and uterus are shown. The samples, with or without previous PK treatment, were deglycosylated with PNGase F. Membranes were probed with the mAbs 3F4 and 2301 as indicated. **A:** The brain has relatively large amounts of full-length isoform and PrP^res^ C2 fragment but the N-terminus truncated PrP^C^ fragment (C1) is poorly represented. In addition, brain preparation shows a previously unreported PK-sensitive fragment with molecular weight of 25 kDa (arrow), detectable only in deglycosylated samples, of undetermined origin. **B:** The C1 fragment is relatively better represented in the pituitary gland **C:** while it is overly abundant in the uterus.

Notably, the antibody 2301 to the PrP C-terminal region revealed that in contrast to findings in the brain, most of the PK-sensitive PrP had the electrophoretic mobility of approximately 18 kDa (after deglycosylation) in all non-neural tissues, whereas the full length isoform appeared to be underrepresented (Fig. 4, data not shown). This finding was particularly prominent in the uterus but least evident in the pituitary gland (Fig. 4). Epitope mapping indicated that the 18 kDa fragment was truncated at the N-terminus matching the characteristics of the fragment identified as C1 (Fig. 4) [31]. In addition to being PK-sensitive, C1 could be easily distinguished from the unglycosylated form of PrP^res^ detectable in PK untreated samples, named C2, which electrophoretically migrated to 19 kDa as in other organs (Fig. 4).

## Discussion

Our study confirms the diagnosis of vCJD in the present case, based on the characteristics of the PrP^res^ and the methionine homozygosity at codon 129 of the PrP gene, the last feature being invariably present in vCJD [32]. However, we also observed two unusual features in this case. The first is the long disease duration of 32 months, which is more than twice the 14 month mean duration of the British cases of vCJD [3]. However, cases of up to 40 months duration after the diseases onset have been reported [3], [33]. The second unusual feature is the absence of typical spongiform degeneration which likely stemmed from the long duration of the disease. The long disease duration likely led to extensive loss of neurons, in which most of the vacuoles are formed, with ensuing astroglial scar [34].

As previously reported [21], the BSE exposure most likely occurred between the early eighties, when the BSE epidemic emerged in the UK, and 1992, when the patient immigrated to the US. This assumption is consistent with an incubation period of 9 to 21 years, which correlates well with the medium incubation period of 17 years estimated for the UK cases of vCJD [35].

The brain PrP^res^ of the present case displayed the glycoform ratio and electrophoretic mobility characteristic of the PrP^res^ associated with vCJD [5]. One exception is the cerebellum where the monoglycosylated and unglycosylated PrP^res^ isoform migrated slightly faster than the PrP^res^ from other brain regions and resolved in three bands. The variation in PrP^res^ electrophoretic characteristics between the cerebellum and the cerebral cortex is not surprising for it has also been observed in sCJD [36]. Yet to our knowledge it has never been reported in vCJD. Finally, contrary to previous reports [29], PrP^res^ type 1 did not co-occur with type 2. This discrepancy might stem from our rigorous PrP digestion with PK and from the use of different antibodies, an approach that rules out the possibility that partially cleaved fragments derived by the incomplete digestion of PrP^Sc^ be misinterpreted as the type 1 fragment [30], [37].

The major finding of the present study is the demonstration that PrP^res^ is present in a number of non-CNS tissues and organs which previous studies had reported as free of PrP^res^ (Table 1 and 2) [14]–[19, P. Brown, unpublished data]. These tissues include the dura mater, skin, liver, kidney, pancreas, descending colon, uterus and ovary (Table 2 and Fig. 3). The use of NaPTA, along with the long disease duration, may both have contributed to the undisputed detection of PrP^res^ in these organs in this case. The glycoform ratio of the brain PrP^res^ was not retained in every peripheral organ examined (Fig. 4). In the pituitary gland and the skin the diglycosylated and monoglycosylated PrP^res^ isoforms were about equally represented thus the diglycosylated isoform was not dominant. On the other hand, electrophoretic mobility appeared to match that of the brain. Variations in the glycoform ratio could be assessed only on the TH because the glycoform ratio, as well the electrophoretic mobility, is affected by NaPTA enrichment [14].

The presence of prion in the human dura mater is not surprising because sCJD has been transmitted following transplantation of dura obtained from sCJD-affected cases [38]. However, to our knowledge this is the first immunoblot demonstration of PrP^res^ in the dura mater in any prion disease. The detection of relatively large amounts of PrP^res^ in the dura mater raises the possibility of contamination with brain tissue at autopsy. Although this possibility cannot be completely ruled out, extensive rinses in PBS were performed before homogenization in some experiments without observing a reduction in the amount of the PrP^res^ detected.

Prion infectivity of kidney and liver has been demonstrated by bioassay in other human prion diseases [39], and PrP^res^ has been observed in the kidney of scrapie infected sheep [40]. The presence of PrP^res^ has also been reported in kidney, liver and pancreas of scrapie infected mice in association with lymphofollicular proliferation [41]. This last finding is relevant to the present case in which multiple lymphocytic infiltrates with follicular pattern were present in the kidney. However, contrary to this report, we observed no significant inflammatory reaction in any of the other tissues which contained PrP^res^. A puzzling finding of our study is the presence of PrP^res^ albeit in small amounts in the kidney but not in the urinary bladder. This apparent discrepancy is relevant to the recent demonstrations of prion infectivity in urine of animals carrying experimental or naturally occurring prion diseases [42]–[46]. It would indicate that prion infectivity in urine is acquired from the kidney while the urinary bladder acts as a bystander. However the amount of PrP^res^ we observed in the kidney was minimal, and might have not been sufficient to infect the urine and to propagate to the bladder in detectable amounts. Indeed we failed to demonstrate PrP^res^ in the urine in the present case even after hundred-fold urine concentration (data not shown). Obviously more studies are needed to clarify this issue.

The present study also demonstrates for the first time the presence of PrP^res^ in the skin in a human prion disease. Previously, PrP^res^ has been detected in the skin from animals with experimental or naturally occurring scrapie [47] as well as in the antler velvet of elk affected by CWD [48].

Furthermore, it is remarkable that we observed PrP^res^ in the uterus and the ovary, a finding which implicates the reproductive system, thereby raising the possibility of maternal transmission of vCJD. Vertical transmissibility of prion infection has been demonstrated in transgenic mice infected with BSE [49]. Related literature on human prion diseases is very scanty. Pregnancy completed to delivery has been reported in sCJD, iatrogenic CJD and vCJD [50], [51]; however, transmission to the progeny has not been examined in detail or confirmed in any of these cases. The first detailed determination of PrP^C^ and PrP^res^ in the reproductive and gestational tissues from a sCJD patient has been carried out only recently [51]. Although this study failed to detect PrP^res^, remarkably it showed that, in uterine tissue obtained at biopsy, most of the PK-sensitive PrP is truncated at the N-terminus and matches the C-terminal PrP^C^ fragment C1 which is generated during normal PrP^C^ metabolism [51]. Similarly, in the present case we observed that the C1-like fragment was largely predominant over the full-length PrP^C^ in the uterus, and it was easily digested by PK but it was present along with a significant amount of characteristic vCJD PrP^res^ (Fig. 4). Since the N-terminus of the PrP^res^ type 2 associated with vCJD is at residues 92–99, the uterine PrP^res^ must have formed from the full length PrP^C^ rather than from C1, the N-terminus of which is at residues 111–112 [31], [52]. These findings raise the question of the origin of the PrP^res^ found in the uterus, a question that is currently unanswered. A similar question may be raised for the urine, in which although the prion infectivity has been demonstrated in animals by bioassay [42]–[46], the only detected form of PrP under normal condition in animals and humans, is a fragment matching the C1 [53], [54, Notari et al., unpublished data].

All these considerations notwithstanding, the widespread presence of PrP^res^ in visceral organs that we observed in the present case further reinforces the concerns over iatrogenic transmission of vCJD. These concerns are already compelling given the multiple reports of vCJD transmission by blood transfusion.
